# Accurate and Precise DNA Quantification in the Presence of Different Amplification Efficiencies Using an Improved *Cy0* Method

**DOI:** 10.1371/journal.pone.0068481

**Published:** 2013-07-08

**Authors:** Michele Guescini, Davide Sisti, Marco B. L. Rocchi, Renato Panebianco, Pasquale Tibollo, Vilberto Stocchi

**Affiliations:** Department of Biomolecular Sciences, University of Urbino “Carlo Bo” Via I Maggetti, Urbino, Italy; Institute of Molecular Genetics IMG-CNR, Italy

## Abstract

Quantitative real-time PCR represents a highly sensitive and powerful technology for the quantification of DNA. Although real-time PCR is well accepted as the gold standard in nucleic acid quantification, there is a largely unexplored area of experimental conditions that limit the application of the *Ct* method. As an alternative, our research team has recently proposed the *Cy0* method, which can compensate for small amplification variations among the samples being compared. However, when there is a marked decrease in amplification efficiency, the *Cy0* is impaired, hence determining reaction efficiency is essential to achieve a reliable quantification. The proposed improvement in *Cy0* is based on the use of the kinetic parameters calculated in the curve inflection point to compensate for efficiency variations. Three experimental models were used: inhibition of primer extension, non-optimal primer annealing and a very small biological sample. In all these models, the improved *Cy0* method increased quantification accuracy up to about 500% without affecting precision. Furthermore, the stability of this procedure was enhanced integrating it with the SOD method. In short, the improved *Cy0* method represents a simple yet powerful approach for reliable DNA quantification even in the presence of marked efficiency variations.

## Introduction

Real-time quantitative polymerase chain reaction (real-time PCR) is the method of choice for absolute or relative quantification of nucleic acids (DNA and RNA if preceded by the reverse transcription step) because of its rapidity, accuracy and sensitivity [1]–[3]. Although real-time PCR is well accepted as the gold standard in nucleic acid quantification and is widely used for validating the results of large-scale microarray experiments [4], two key issues, namely data quality assurance and proper data analysis, may compromise the acquisition of reliable biological results [5]–[7].

The widely accepted quantification method for determining the quantification cycle (Cq) in real-time PCR is the *Ct* (threshold cycle) method. The *Ct* value is defined as the fractional cycle number in the log-linear region of PCR amplification in which the reaction reaches fixed amounts of amplicon DNA [8]. This method requires generating serial dilutions of a given sample and performing multiple PCR reactions on each dilution. The threshold-cycle values are then plotted versus the Log of the dilution and a linear regression, from which the mean efficiency can be derived, is performed [9]. This approach involves the comparison of identical efficiency among amplifications. PCR efficiency can be defined as the fold change in the amount of amplified DNA after each cycle of PCR. In an ideal PCR, the DNA template should double at each cycle according to the equation:
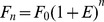
(1)where *n* is the cycle number, *F_0_* is the base fluorescence value and efficiency (*E* = 1) is constant and maximal over the entire reaction. However, empirical evidence shows that *E* is not always 1 and constant, conversely it progressively declines at the building up of products and consuming of reagents [10], [11]. Hence, we can distinguish *E* in *E_max_* defined as the maximal initial value of PCR efficiency, *E_n_* is the efficiency value at the *n* cycle and the rate of loss in *E* after each cycle is defined as *ΔE*. To provide a complete picture we will discuss overall efficiency when describing the global efficiency of the amplification system calculated using the standard curve method [8]:
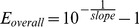
(2)where the slope is calculated from the linear regression between the Log of the initial DNA template and the Cq.

Small amplification variations may occur in any PCR quantification potentially leading to estimate uncertainty. Among the numerous factors that affect the sensitivity, accuracy, and reliability of real-time PCR assay, are many substances found in biological samples such as co-extracted contaminants, which can inhibit PCR, confounding template amplification and analysis [12]–[15].

Furthermore, poor quality primers or difficult samples may lead to sub-optimal amplification. Imperfect amplification primers may be the result not only of non-optimal primer design but, sometimes, primer sequence and/or position are constrained, as in the case of detection of sequence variants, intron spanning primers etc. [16]. Sub-optimal amplification may also be the result of the inhibiting agents used during nucleic acid extraction or co-purified components from the biological sample such as bile salts, urea, haeme, heparin, and immunoglobulin G. The presence of inhibitors results in deviations of the amplification kinetic from the optimal amplifications obtained using standard samples [17].

For these reasons, many tools, based on amplification efficiency, have been developed to detect outlier amplifications [18]–[20]. A quality test tool, called SOD, has also recently been developed. SOD is not based on direct amplification efficiency estimation in order to detect outliers, but relies on monitoring the shape of the amplification curve [21].

The most common procedure used to account for any differences in PCR efficiencies among samples is to amplify a reference gene in parallel with the reporter gene and to relate their expression levels. However, this approach assumes that the two assays are inhibited to the same degree, which is not always the case. Such variations in assay inhibition are a particular problem in absolute quantification, where an external calibration curve is used to calculate the number of transcripts in the test samples, an approach that is commonly adopted for quantification of pathogens [22].

In the last few years, a number of studies have attempted to address these problems determining reaction amplification efficiency through the application of different mathematical models [17], [23]–[28]. However, the key issue is that the exact equation of the PCR amplification kinetics is still elusive, and the proposed models are only good approximations of single portions of the amplification kinetic.

Bearing in mind these concerns, our group proposed an alternative method (*Cy0* method), in which a new quantitative entity, *Cy0*, is defined. The *Cy0* method is similar to *Ct*, but it offers the important advantage of taking into account the kinetic parameters of the amplification curve and may compensate for small variations among the samples being compared [29]. Conversely, with a marked decrease in amplification efficiency the *Cy0* method underestimates. Our proposed improvement in the *Cy0* method is based on the use of kinetic parameters calculated in the curve inflection point to compensate for efficiency variations. In addition, the enhanced *Cy0* method was integrated with SOD analysis to quantify starting DNA quantity in the presence of different amplification kinetics due to inhibition of primer extension and imperfect primer design. The advantages of the improved *Cy0* method were then evaluated in mtDNA quantifications from skeletal muscle samples obtained from fine needle aspiration [30].

## Materials and Methods

### Quantitative Real-Time PCR

The DNA standard consisted of a pGEM-T (Promega) plasmid containing a 104 bp fragment of the human mitochondrial gene NADH dehydrogenase 1 (MT-ND1) as insert. This plasmid was purified using the Plasmid Midi Kit (Qiagen) according to the manufacturer’s instructions. The final concentration of the standard plasmid was estimated spectophotometrically by averaging three replicate A_260_ absorbance determinations.

This DNA fragment was amplified for quantification by the high amplification efficiency (HE) primer pair (forward ND1F2∶5′-ACGCCATAAAACTCTTCACCAAAG-3′ and reverse ND2∶5′-TAGTAGAAGAGCGATGGTGAGAGCTA-3′) and low amplification efficiency (LE) primer pair (forward ND1F5∶5′-ATAAAACTCTTCACCAAAGAG-3′ and reverse ND2∶5′-TAGTAGAAGAGCGATGGTGAGAGCTA-3′).

Real-time PCR amplifications were conducted using LightCycler® 480 SYBR Green I Master (Roche) according to the manufacturer’s instructions, with 500 nM primers and a variable amount of DNA standard in a 20 µl final reaction volume. Thermocycling was conducted using a LightCycler® 480 (Roche) initiated by a 10 min incubation at 95°C, followed by 40 cycles (95°C for 5 s; 60°C for 5 s; 72°C for 20 s) with a single fluorescent reading taken at the end of each cycle. Each reaction combination, was performed in 4–6 replicates and all the runs were completed with a melt curve analysis to confirm the specificity of amplification and lack of primer dimers.

### Muscle Fine Needle Aspiration (FNA)

Students affiliated with the University of Urbino took part in this study. Individuals were considered eligible if they were *>*18 years old. Written informed consent was obtained from all participants. Ethical approval for this study conformed to the standards of the Declaration of Helsinki. The Urbino University Ethical Committee (Approval Number 28507) approved the protocol before study initiation.

Skeletal muscle was obtained by fine needle aspiration (FNA) from the vastus lateralis muscle. Muscle FNA was performed with a 22-G spinal needle (Becton Dickinson, Madrid) under ultrasound guidance as previously described by Guescini et al. [30]. Muscle tissue was rapidly thawed and incubated with 0.2 mg/ml of Proteinase K at 55°C for 10 min. Subsequently, genomic DNA and total RNA were co-purified using silica micro columns by the RNeasy Micro Kit (Qiagen) according to the manufacturer’s instructions. Real-time PCR amplifications were conducted using Specific primers for MT-CO1 (forward: 5′-GTGCTATAGTGGAGGCCGGA-3′ and reverse: 5′-GGGTGGGAGTAGTTCCCTGC-3′). and ATP6 (forward: 5′-ATGAGCGGGCACAGTGATTA-3′ and reverse: 5′-AGGGAAGGTTAATGGTTGATA-3′) in a Light-Cycler 480 SYBR Green I Master (Roche, Basilea, Switzerland) according to the manufacturer’s instructions, with 300 nM primers and 2 µl of purified FNA sample in 20 µl of final reaction volume. Thermocycling was performed using a LightCycler 480 (Roche) initiated by a 10 min incubation at 95°C, followed by 40 cycles (95°C for 5 sec; 60°C for 5 sec; 72°C for 10 sec) with a single fluorescent reading taken at the end of each cycle. Each reaction was conducted in duplicate and completed with a melt curve analysis to confirm the specificity of amplification and lack of primer dimers.

### *Cy0* Method

The *Cy0* value [29] is the intersection point between the abscissa axis and tangent of the inflection point of the Richards curve obtained by the nonlinear regression of raw data. The Richards equation is an extension of the symmetric logistic-type growth curve; specifically, when *d* coefficient is equal to 1, the symmetric logistic-type and Richards (asymmetric logistic-type) curves are the same. Shape of amplification curves in real-time PCR ranges from a perfect symmetric to a strongly asymmetric shape, for example in presence of inhibitors [21]. The *Cy0* method was performed by nonlinear regression fitting of the Richards function [31], in order to fit fluorescence readings to the 5-parameter Richards function.
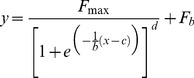
(3)Where *y* is the reaction fluorescence at cycle *x*, *F_max_* is the maximal reaction fluorescence, *b* can be considered a slope parameter, *c* is the transition parameter and *d* represents the Richards coefficient, and *F_b_* is the background reaction fluorescence. The five parameters that characterized each run were used to calculate the *Cy0* value by the following equation, corrected in order to eliminate the influence of baseline correction *(F_b_). Cy0* values were obtained as follows:



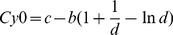
(4)When the flex ordinate of the raw data curve is lower than *F_max_/e* (where *e* = 2.718281…), the Richards curve does not yield a good fit because it is not possible to estimate the parameter *d*; in this case, the upper points are progressively removed, until an acceptable fitting is achieved [32]. *Cy0* was calculated using the following web interface: http://www.cy0method.org/, specifically developed by the authors of this article.

### SOD Calculation

Shape based kinetic outlier detection (SOD) was based on the shapes of the amplification curves. In order to fit fluorescence raw data, nonlinear regression fitting of 5-parameter Richards function was performed. The following shape parameters were used: the plateau value of the amplification curve (*F_max_*), the tangent straight line slope in the inflection point (*m*) and the y-coordinate of the inflection point (*Y_f_*). For further details, see Sisti et al. [21].

All other statistical elaborations and graphics were obtained using specific VBA macro developed in the MS Excel and Statistical Packages for Social Sciences (SPSS 13.0).

## Results and Discussion

### Mathematical Model for *Cy0* Correction

Regarding the kinetic of PCR amplification we considered the logistic model as previously reported by Chervoneva *et al.* and Rutledge & Stewart [33], [34].
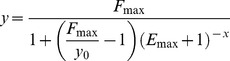
(5)where *F_max_* is the height of amplification profile, *E_max_* is the starting maximal efficiency value of curve growth rate and y_0_ is the initial amount of DNA template. By a simple mathematical elaboration (reported in Data File S1), it is possible to determine the curve slope of the inflection point as:
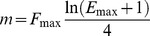
(6)subsequently, using this equation it is possible to calculate the value of the starting reaction efficiency:



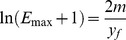
(7)This relation shows that the slope of the inflection point, scaled for its ordinate, is the natural logarithmic value of starting efficiency (*E_max_*+1).

Starting from two amplification reactions (A and B) showing the same starting DNA template (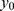), but different *E_max_*, we can consider the following equivalence:
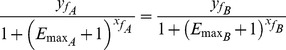
(8)from which, introducing some useful approximations and transformations (see Data File S1), it is possible to formulate the following equation:
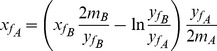
(9)where xf and yf are the x- and y-coordinates of the inflection point of the amplification curve and m is the slope of the curve in inflection point. In this equation the xf values represent the Cq of the two amplification reactions while yf and m account for the kinetic parameters of the amplification (Emax and ΔE). In particular, in the presence of different amplification kinetics for curves A and B, the Cq of curve B (xfB) can be overlapped to the Cq of curve A (xfA) applying Eq. 9. Notably, to perform this procedure the only parameters needed are xf, yf and m of the two curves.

The proposed procedure refers to the comparison of two amplification curves; however, this formula can be easily generalized to the standard curve quantification method. In fact, we propose using 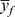 and 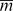 as reference values calculated as the arithmetic mean of each *y_fi_* and *m_i_*, estimated from all standard amplification curves, and as Cq, we replaced *x_f_* with *Cy0*. After profile correction, the two curves become parallel justifying the use of the *Cy0* value as Cq. Hence, in the presence of unknown amplification profiles significantly different in shape from that of the standard curve the obtained *Cy0* value should be corrected using the following formula:
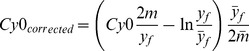
(10)


### Assessing the *Cy0* Correction Model in Theoretical Logistic Curves

The proposed *Cy0* correction provides for: *a)* curve shift of a factor equal to [ln(*y_fB_*/*y_fA_*)]/(*y_fB_*/2*m_A_*)] and *b)* scaling on the abscissa axis using the ratio (*m_B_*/y_fB_*y_fA_/*m_A_*). It should be noted that all these procedures were not necessarily applied simultaneously. Their application depends on the characteristics of the curves to be compared and consequently Eq. 10 progressively becomes simpler when one of the standard curve parameters (*m or y_f_*) matches with one of the corresponding parameters of the unknown curve.

**Figure 1:**
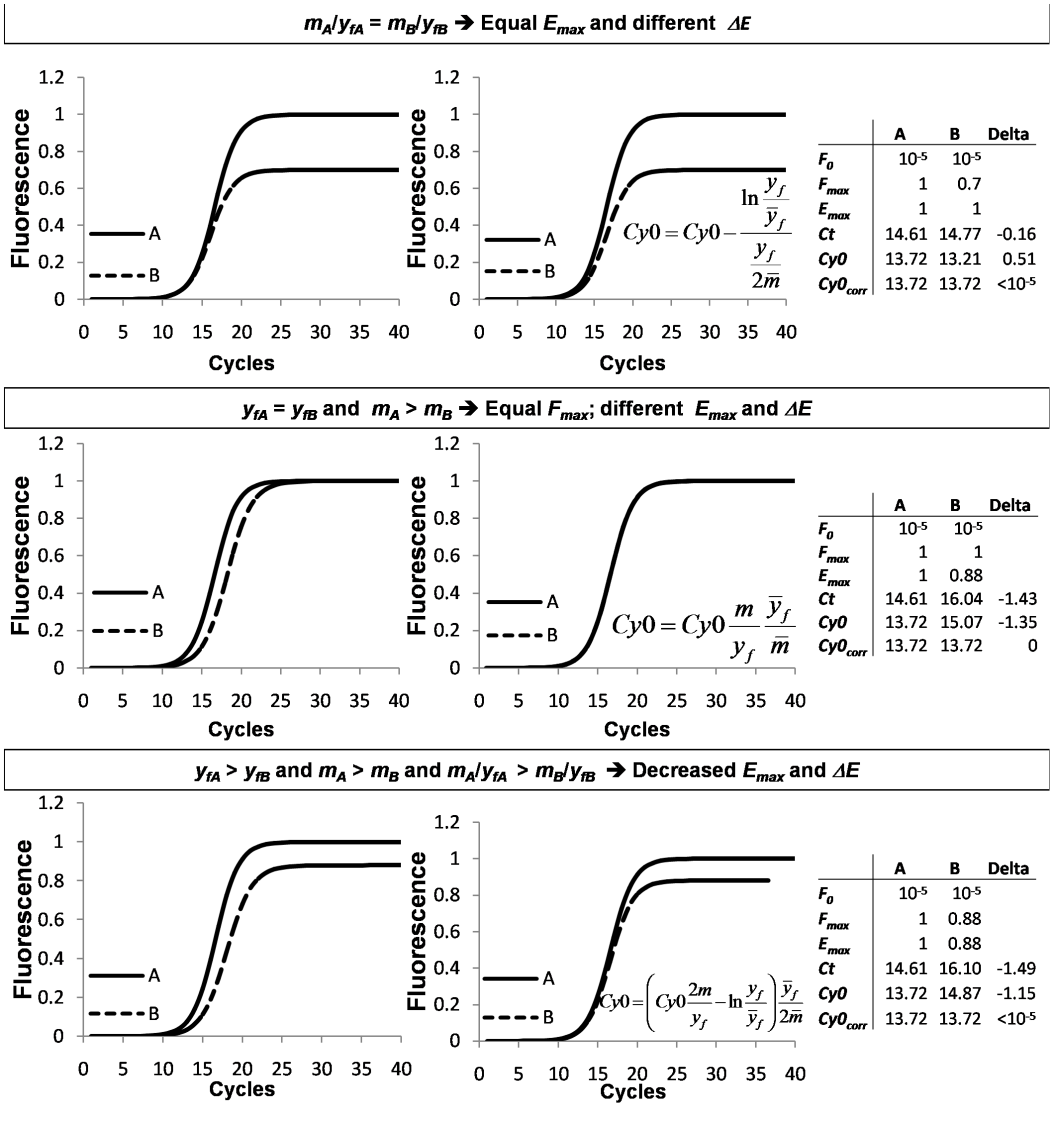
Simulation of amplification curves with different kinetics using logistic equation. Left plots show the starting fluorescence profiles A and B, in which A represents the optimal amplification while B refers to the non-optimal amplification profile. Right plots report the amplification profiles after application of proposed correction. In these analyses, we used profile A as a reference, hence the A profiles always remain the same while the B profiles were corrected using the formula reported near the plots. All the reported formulas derive from Eq. 10. In an attempt to explain the components of this equation, we deleted the terms that nullify according to the differences and overlappings in the amplification profiles. The inner tables show the kinetic parameters of the fluorescence profiles and the Cq calculated as *Ct*, *Cy0* and *Cy0_corr_* and differences among them are also shown.

In an attempt to demonstrate the performance of the proposed *Cy0* correction, using Eq. 5, we created two amplification profiles characterized by different *F_max_* and *E_max_* and hence different *ΔE* [11], but the same initial DNA template (*y_0_*). The analysis of the resulting profiles allowed us to show how the *Cy0* correction worked and to explain the shifting and scaling factors used in Eq. 10. In these analyses we showed two fluorescence profiles: A and B; curve A represented the optimal condition, whilst curve B represented a non-optimal fluorescence profile (Fig. 1 left graphs). After correction, the fluorescence profile of curve B was modified following the proposed procedure using the parameters of curve A as a reference (Fig. 1 right graphs) and *Cy0* was recalculated (*Cy0_corr_*).

In the first case, the two curves were characterized by equal *E_max_* (*E_max_* = 1) and different *F_max_* corresponding to 1 and 0.7, respectively (Fig. 1 upper panels). In these conditions, profile B was less steep and slightly shifted towards the right compared to profile A. The *Cy0* values obtained from the two curves differed, and in particular, the *Cy0* value calculated from the curve with lower *F_max_* slightly overestimated the input DNA quantity, whilst the *Ct* underestimated the starting quantity. As we can observe in the inner table, after *Cy0* correction, the error became very small (*Cy0_A_*-*Cy0_corr-B_* <10^−5^). Then, we created two profiles where *F_max_* was equal but *m* differed, as shown in Figure 1 (middle panels) Curve B (*E_max_* = 0.88) was significantly more delayed than curve A producing a marked underestimation using both *Cy0* and *Ct* methods. After the application of the proposed correction, curves A and B overlapped resulting in a full correction of the initial DNA estimate. Finally, we provided an example that summarizes the two previous cases, in this elaboration two profiles differing in both *E_max_* and *F_max_* were created, and in this case as well the proposed correction allowed us to compensate for these differences (Fig. 1 lower panels). Hence, we can conclude that *Cy0* correction as reported in Eq. 10 can be applied in all conditions.

The presented data showed that theoretically, it is possible to account for differences in kinetic parameters between two amplification profiles estimating *x_f_*, *y_f_* and *m.*

The improved *Cy0* method re-proposes two previously introduced concepts: curve normalization on *F_max_* [35] and Cq correction based on the value of the slope in the maximal of the second derivative [36]. These aspects were incorporated in the proposed method and further developed starting from the logistic curve. Specifically, the significant differences consisted in the use of *y_f_* for normalization to avoid bias due to asymmetric amplifications and the weighting of the slope of the curve on the *y_f_* value. Furthermore, to the best of our knowledge, the introduced shift factor [ln(*y_fB_*/*y_fA_*)]/(*y_fB_*/2*m_A_*)] has never been reported in literature.

### Low and High Amplification Efficiency Systems

**Figure 2:**
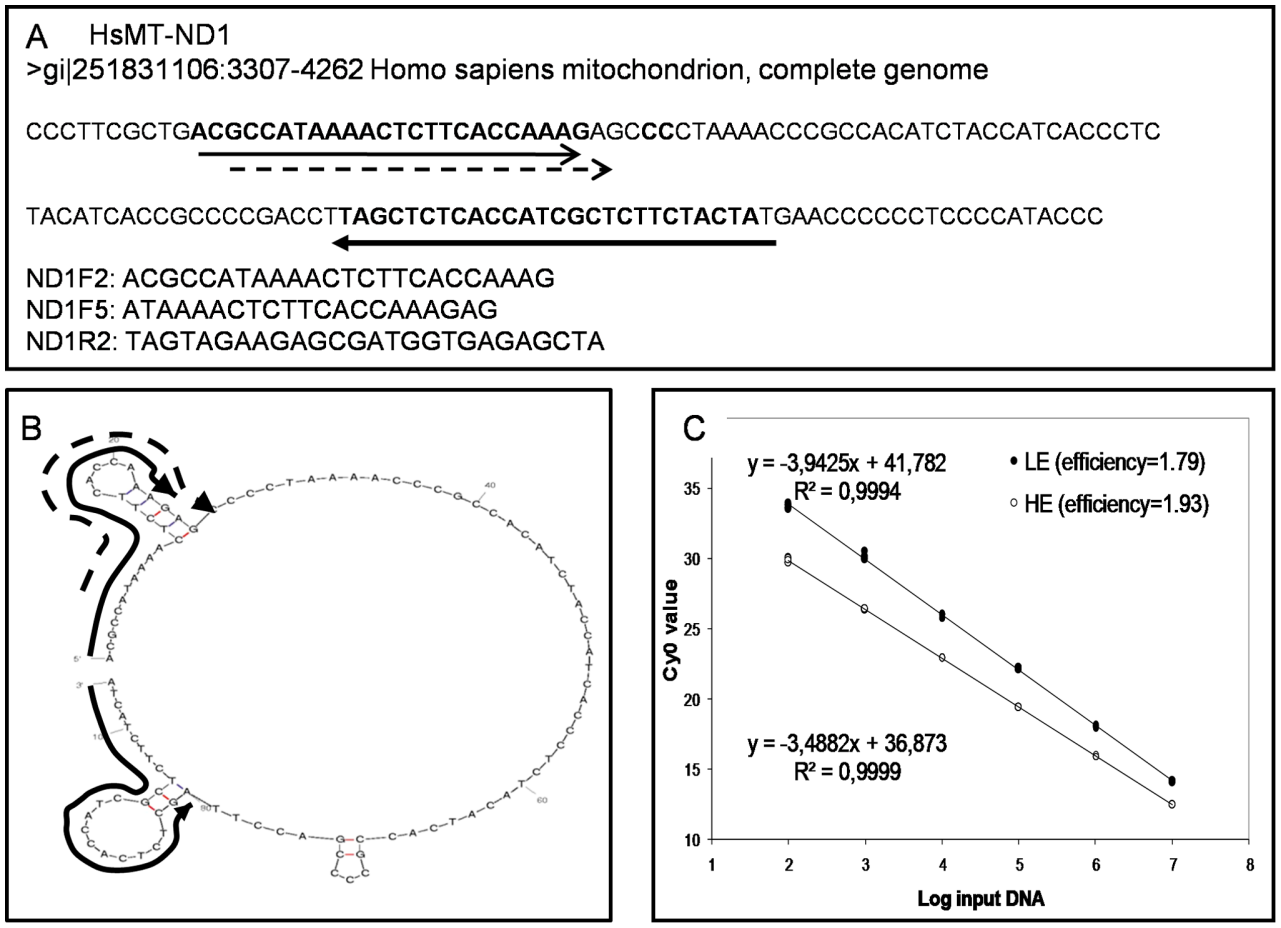
Description of the High Efficiency (HE) and Low Efficiency (LE) systems. The HE and LE systems were obtained amplifying the mitochondrial MT-ND1 gene with the same reverse primer ND1R2 but using ND1F2 or ND1F5 as forward primer, respectively. Panel (A) shows the primer annealing locations in the mitochondrial sequence; panel (B) shows the secondary structure of the obtained amplification products calculated using http://mfold.rna.albany.edu/. This analysis shows that ND1F5 primer overlaps the stem-loop at 5′ terminus, The overlapping coupled with its reduced length impairs the primer’s ability to bind the DNA template. Panel (C) shows the overall efficiency analysis of the two systems (HE and LE). The slopes of the two regressions are significantly different, with slope_HE_ = −3.488; slope_LE_ = −3.942; consequently, the overall efficiencies, calculated using the equation 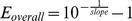, were *E_overall-HE_* = 0.93; *E_overall-LE_* = 0.79.

To examine the usefulness of the developed quantification method in compensating for PCR efficiency, two amplification systems were used. The first is characterized by a high amplification efficiency (HE; Fig. 2C black symbols), while the second showed a low amplification efficiency (LE; Fig. 2C white symbols) (Fig. 2). The two systems were obtained amplifying the same DNA sequence with the same reverse primer ND1R2 but using ND1F2 or ND1F5 as forward primer for the HE and LE systems, respectively. Specifically, the annealing region of the primer ND1F5 is shifted only 5 nucleotides downstream from the ND1F2 primer location and shortened by three nucleotides (Fig. 2A). In this region, the DNA template shows a stem-loop secondary structure (evaluated using http://mfold.rna.albany.edu/results/10/12Aug30-10-05-01/) that competes with the forward primers for annealing (Fig. 2B). The primer ND1F5 is probably less effective than ND1F2 in template binding. Using this strategy, we were able to amplify the same DNA input template with two amplification systems that work with marked efficiency differences. The amplification efficiencies of the HE and LE systems were determined by the standard curve method. The obtained standard curves and the corresponding regression equations are shown in Fig. 2C and the obtained overall efficiencies of HE and LE were *E_overall-HE_* = 0.93 and *E_overall-LE_* = 0.79.

### Evaluation of the Correction Model in the Presence of Decreased Amplification Efficiency

Hence, we investigated the possible application of this procedure to experimental amplification plots obtained by the HE and LE systems (Fig. 2). The LE system is clearly far from a logistic profile, hence this allowed us to evaluate the robustness of the proposed model in asymmetric amplifications that deviate from a logistic shape.

**Figure 3:**
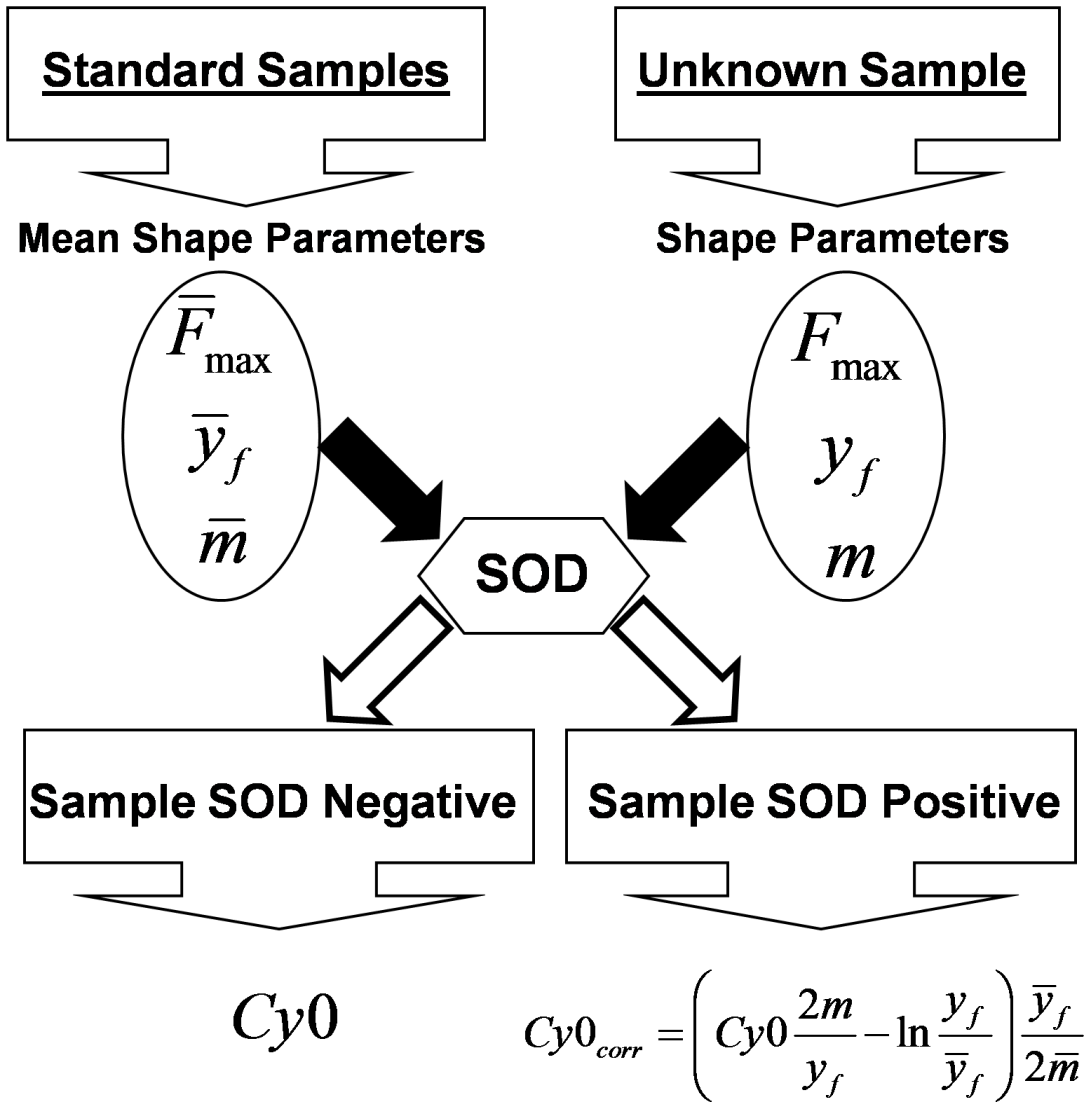
Flowchart of the integration between *Cy0* and SOD methods and application of the *Cy0* correction. *F_max_*, *y_f_* and *m* are the shape parameters corresponding to each unknown amplification curve. These parameters are compared with mean shape parameters (marked above) calculated as the mean of the single parameters of all the standard curves.

**Figure 4:**
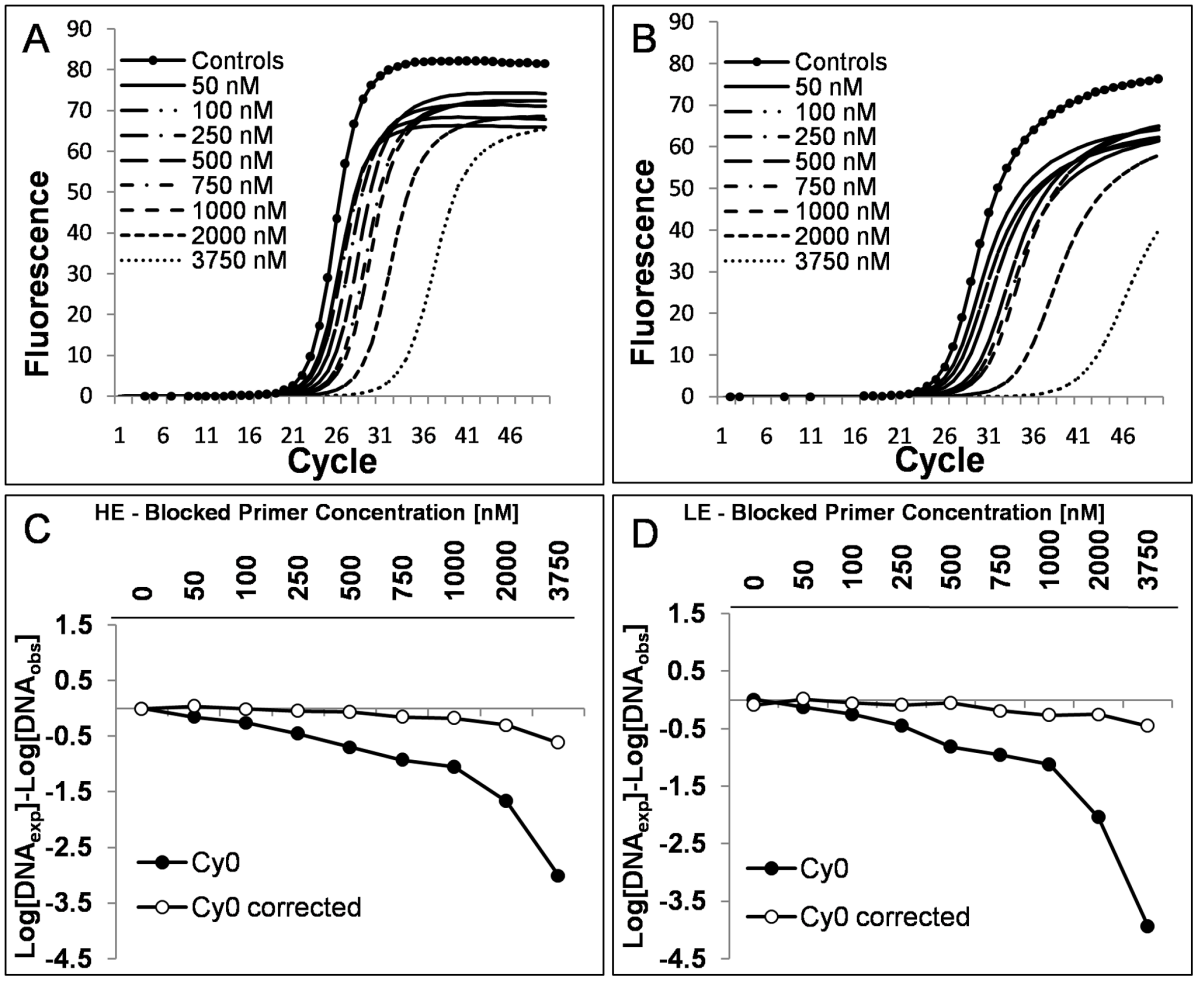
Effect of increasing PCR inhibition on the accuracy of the HE and LE amplification systems. PCR quantifications were performed using HE and LE systems in the presence of an equal starting number of template molecules and increasing blocked primer concentrations. Panels (A) and (B) show the obtained amplification plots for each system. Panels (C) and (D) show the relative errors obtained using *Cy0* and *Cy0* corrected following Eq. 10. The relative error was reported as *Log(DNA_exp_)*–*Log(DNA_obs_)* where *Log(DNA_exp_)* is the number of expected molecules and *Log(DNA_obs_)* is the number of calculated molecules using the *Cy0 (Black dots)* or *Cy0_corrected_ (White dots).* Each symbol represents a mean 6 runs. SOD detected almost all the runs in which the blocked primer was added as outliers.

**Figure 5:**
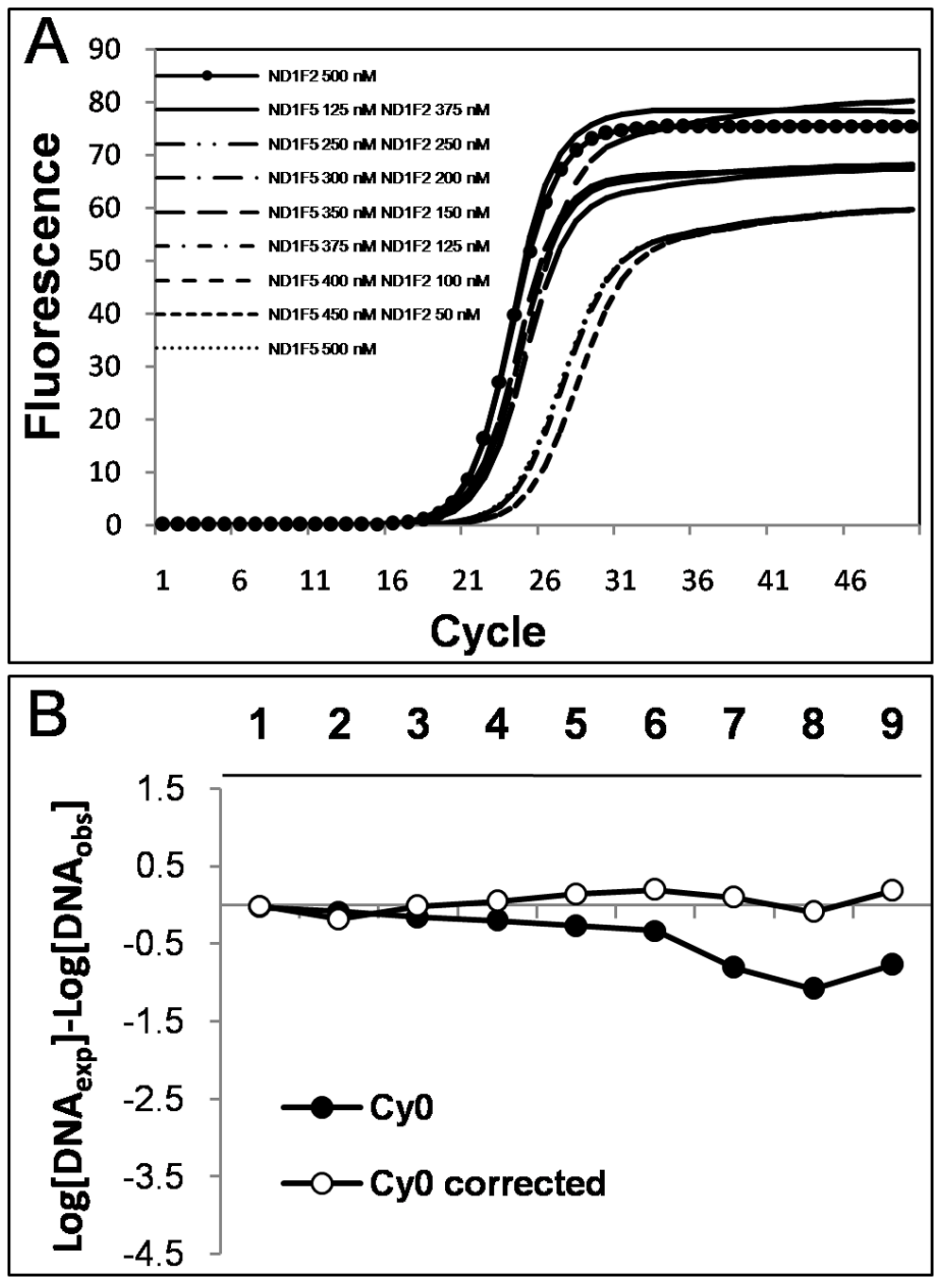
Real-time PCR quantifications in the presence of primers with different annealing abilities. Panel (A) shows amplification plots obtained for each condition; (B) shows the relative errors *Log(DNA_exp_)*–*Log(DNA_obs_)* obtained using *Cy0* and *Cy0* corrected following Eq. 10. PCRs were performed in the presence of an equal starting number of template molecules by using 500 nM of ND1R2 as reverse primers and different combinations of ND1F2 and ND1F5 as forward primers. Specifically, the PCRs were set as follows: 1) 500 nM ND1F2; 2) 375 nM ND1F2 and 125 nM ND1F5; 3) 250 nM ND1F2 and 250 nM ND1F5; 4) 200 nM ND1F2 and 300 nM ND1F5; 5) 150 nM ND1F2 and 350 nM ND1F5; 6) 125 nM ND1F2 and 375 nM ND1F5; 7) 100 nM ND1F2 and 400 nM ND1F5; 8) 50 nM ND1F2 and 450 nM ND1F5; and 9) 500 nM ND1F5. This experimental model offers the advantage of known PCR efficiencies of extreme conditions (condition 1 corresponds to HE while condition 9 corresponds to LE); hence, we were able to reproduce a gradient of mild decreasing efficiency ranging from 0.93 to 0.79. Black dots represent *Cy0* values while white dots correspond to *Cy0_corrected_* values. Each symbol represents a mean of 4 runs. SOD detected all the runs obtained using [ND1F5]≥250 nM as outliers.

**Figure 6:**
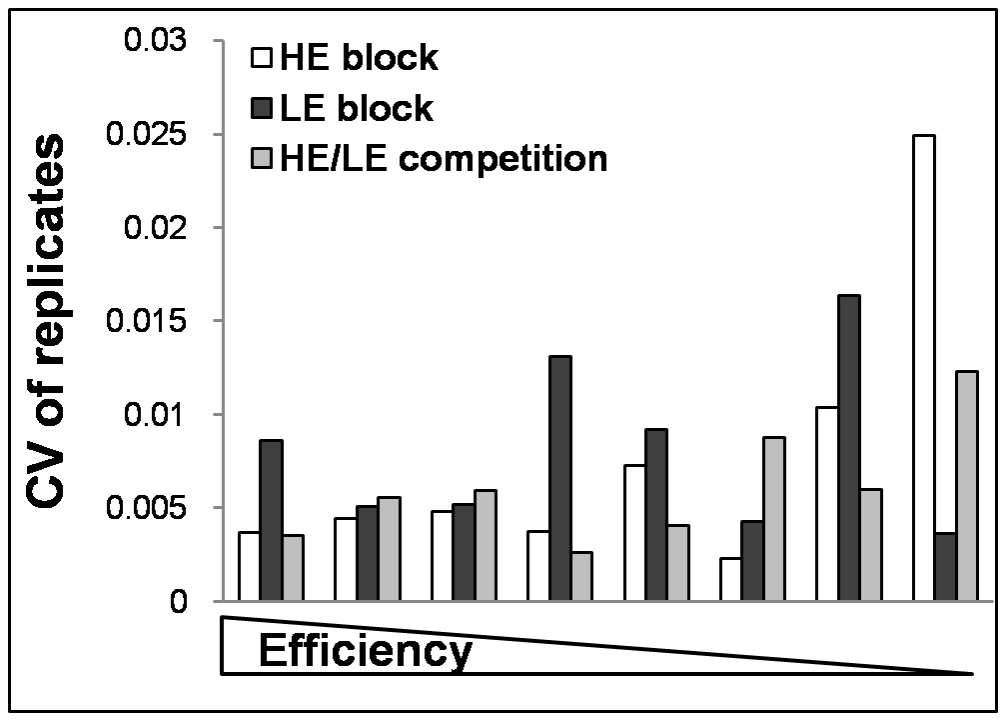
Analysis of the variability among replicates introduced after *Cy0* correction in the studied models. The coefficient of variation was calculated among the corrected *Cy0* values of the replicates corresponding to HE and LE amplifications where blocked primers were added and HE/LE competition.

Correction of the *Cy0* value estimated from an amplification reaction is necessary only in the presence of a marked decrease in the amplification rate compared to that of standard curve samples. In fact, as reported in our previous work, slight efficiency variations are well compensated by the *Cy0* method alone [29]. Hence, we developed a flow chart that integrates the proposed new *Cy0* correction with the previously developed SOD method [21]. As depicted in Figure 3, the first step involves the comparison of the shape parameters (*F_max_*, *y_f_* and *m*) of the unknown amplification with the mean shape parameters (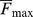,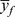 and 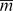) estimated from the standard samples using the procedure reported in Sisti et al [21]. 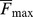,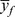 and 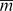 were calculated as the arithmetic mean of all *F_max_*, *y_f_* and *m* values corresponding to each of the amplification curves that make up the standard curve. For SOD negative runs the flow chart leads to the original *Cy0* value whilst the SOD positive values require the application of the proposed *Cy0* correction (Eq. 10) in order to compensate for the difference in amplification efficiency. To test this protocol we used an inhibition model that involved the addition of a blocked primer to the PCR mix. We estimated the inflection point parameters of the amplification curves fitting the Richards equation [29]. We used the Richards curve because it fits very well [28] allowing us to better estimate the inflection point; moreover, it has the advantage of becoming a symmetric logistic-type curve when the Richards parameter *d* = 1 (Data File S2). The blocked reverse primer was progressively added to HE and LE quantification systems resulting in the shift towards the right of the amplification curves (Fig. 4 A and B; efficiencies ranged from 1.93 to 1.67 and from 1.79 to 1.56 for HE and LE systems, respectively) that led to a marked underestimation of input DNA molecules (Fig. 4 C and D). The SOD method efficiently detected almost all the inhibited reactions in which the blocked reverse primer was added. The proposed correction allowed us to decrease the bias in quantification considerably. For example, for the HE system in extreme conditions we obtained Log [DNA_exp_]–Log [DNA_obs_] = −3.01 and −0.62 using *Cy0* and *Cy0_corr_*, respectively. This means that, under these conditions, *Cy0* underestimated the true value by about 1000 fold, whereas *Cy0_corr_* underestimated the value by only 4 fold. Likewise, for the LE system we obtained Log [DNA_exp_]–Log [DNA_obs_] = −3.94 and −0.45 using *Cy0* and *Cy0_corr_*, respectively, corresponding to an underestimation of about 10,000 and 2.5 fold when using *Cy0* or *Cy0_corr_*, respectively. However, one might doubt that such extreme conditions occur often in real-time PCR quantifications. To address this concern, we set up an additional real-time PCR quantification experiment in which the forward primers, ND1F2 and ND1F5, were mixed in different ratios while maintaining the total amount of forward primers constant. This set up mimics a condition in which two primers compete to bind the same template sequence and after primer annealing the polymerization can occur. Hence, using this system we reduced the efficiency of the annealing phase. This setting also offered the advantage of a known amplification efficiency of the extreme conditions (in the presence of only ND1F2 or ND1F5 primers). In these experiments the curve shift was present but it was less pronounced than in the previous experiments. Moreover, as the efficiency of the curves decreased (demonstrated using the standard curve method), the fluorescence profiles showed decreased *F_max_* and steepness, and increased asymmetry (Fig. 5 A; and Data file S3). We used the amplification in the presence of 500 nM ND1F2 and 500 nM ND1R2 as reference (HE system). Once again, SOD correctly identified the runs obtained with ND1F5>250 nM as outliers. The most promising finding showing the effectiveness of the proposed method came to light from the comparison of the extreme conditions, where in the presence of a difference in overall efficiency of 14.18%, we were able to decrease the relative error from 5.89-fold to 0.65-fold using *Cy0_corr_* (Fig. 5 B). Finally, but no less important, we evaluated the variability of the *Cy0_corr_ values* among replicates in the efficiency models used. As expected, these values proved to be very stable with a CV among replicates ranging from 0.002 to 0.02 (Fig. 6).

**Figure 7:**
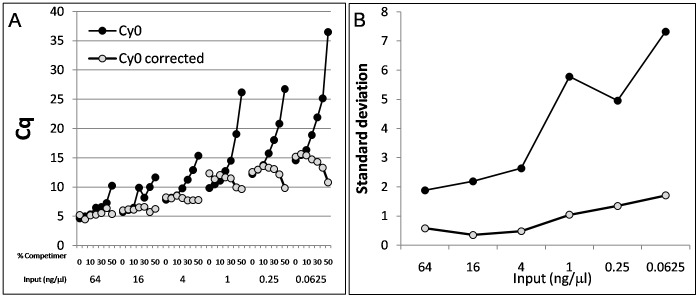
Real-time PCR quantifications in the presence of “Competimer.” The *Cy0* and *Cy0_corrected_* values were calculated from the independent freely available data set named “Competimer”, reported in Ruijter et al. [32]. Briefly, the amplification curves were obtained using decreasing input gDNA amounts (64, 16, 4, 1, 0.25 and 0.0625 ng/µl) and in optimal (% Competimer = 0–5) and inhibited (increasing % Competimer from 10 to 50%) amplification conditions. The 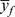 and 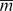 parameters were estimated from optimal amplification conditions. Panel (A) shows *Cy0* and *Cy0_corrected_* values obtained for each condition; Panel (B) shows the standard deviation calculated from the Cqs (*Cy0* and *Cy0_corrected_*) corresponding to the same input cDNA quantity.

To further substantiate the presented results related to *Cy0* method in presence of decreasing amplification efficiency, the *Cy0* correction was applied to the freely available data set named “Competimer”, reported in Ruijter *et al.* [32]. This data set is independent and obtained with completely different chemistry and PCR hardware (Data File S4). The proposed *Cy0* correction allowed us to efficiently quantify the inhibited amplifications as shown by the low Cq shift in presence of increasing % Competimer (Fig. 7A) and by the marked decrease in standard deviation of *Cy0* corrected values compared with uncorrected *Cy0* values (Fig. 7B) in all the conditions tested. These results further demonstrate the effectiveness of the proposed *Cy0* correction and support the notion that it is generally applicable.

### Evaluation of the *Cy0* Correction in Muscle Samples Obtained using Fine Needle Aspiration

**Figure 8:**
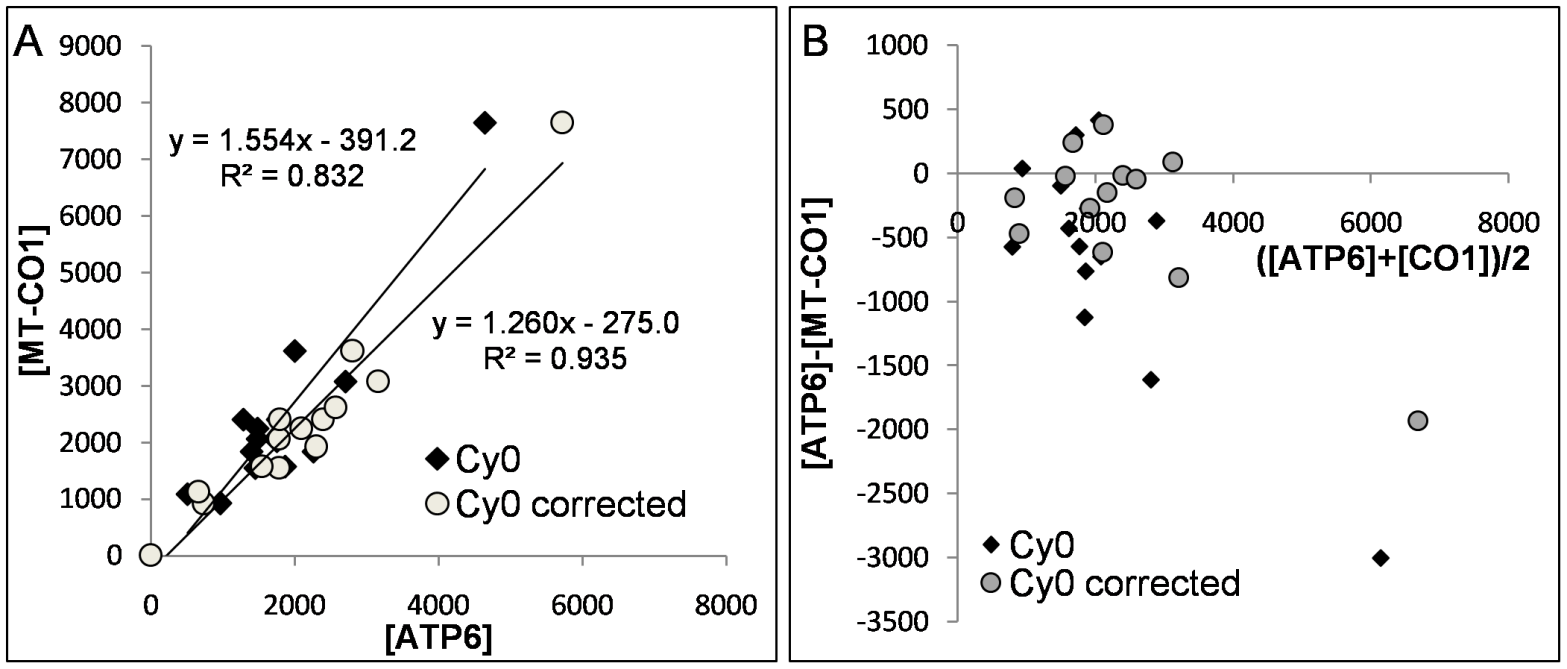
MtDNA real-time PCR quantifications in muscle samples obtained from Fine Needle Aspiration. Mt-CO1 and ATP6 mitochondrial genes were quantified from 40 samples. Only SOD positive samples were included in this analysis. A) shows the descriptive correlation analysis between Mt-CO1 and ATP6 quantification while panel B) shows the Bland-Altman plot.

In order to evaluate the output of the improved *Cy0* method in biological samples, we quantified mtDNA from skeletal muscle fine needle aspiration [30]. This technique allowed us to recover only small quantities of biological materials, hence it was very difficult, if not impossible, to establish PCR efficiency using the standard curve method. To assess the accuracy of the proposed method we estimated the mtDNA quantity by using two amplification systems, one targeted to the MT-CO1 gene and the other to ATP6 gene. Because the mtDNA is a circular DNA molecule, the correlation analysis between MT-CO1 and ATP6 estimated from several specimens should tend to one. The overall MT-CO1 and ATP6 efficiencies, calculated with the standard curve method, using genomic DNA extracted from U87MG cells, were slightly different (*E_overall_* = 0.89; *E_overall_* = 0.94 for ATP6 and MT-CO1, respectively). We analyzed 40 aspirates and for each one MT-CO1 and ATP6 amplifications were performed in duplicate for a total of 160 runs. SOD analysis detected 14 and 5 runs as outliers for ATP6 and MT-CO1, respectively. Interestingly, the amplification system showing the lowest efficiency presented more outlier curves. Subsequently, the correction of the *Cy0* values using the efficiency parameters estimated in the inflection point (Eq. 10) were performed only in the outlier runs. Figure 8A shows the descriptive correlation plot between the MT-CO1 and ATP6 estimates before and after *Cy0* correction. The graph shows that after the proposed correction the slope and the intercept of the correlation straight-line were closer to 1 and 0, respectively; hence, the proposed method enhanced the accuracy of the quantification. The increased precision obtained after correction was shown by the higher *R^2^* value (Fig. 8A). In order to evaluate the agreement between *Cy0* and *Cy0_corr_* outcomes in fine needle aspiration the Bland Altman plot was performed. Using this approach it is possible to show that the quantification differences were proportional to input DNA and that the quantification errors were lower after correction (*p<0.005*; permutation test for paired data). Overall bias in *Cy0* quantification was −25.8% whereas in *Cy0_corr_* the bias was reduced to −10.2%. Specifically, in Figure 8B we can observe that, in outlier runs, uncorrected *Cy0* tended to underestimate the starting DNA and the proposed correction based on kinetic parameters allowed us to account for this.

### Conclusion

Currently, the analysis of real-time PCR data is based almost exclusively on the determination of a quantitative fractional cycle (Cq) value of each sample using the *Ct* method. This value is subsequently referred to a standard curve whose slope estimates overall PCR efficiency.

The *Cy0* method represents a reliable alternative method to calculate a Cq. The advantage of this method stems from the fact that it works very well even in sub-optimal amplification conditions where amplification efficiency may have slight variations among runs [20], [29], [32]. However, in the presence of significant amplification efficiency decrease (in the range 1.60–1.93) the *Cy0* is impaired; hence, determination of amplification efficiency is essential to achieve a reliable quantification.

In low efficiency reactions, ideally, each single quantitative analysis should yield three parameters: a *Cy0* value, as a measure of the number of initial target quantities, an initial efficiency value of the amplification system and an estimate of the rate at which initial amplification efficiency decreases.

The improved *Cy0* method represents a simple but powerful approach to achieve reliable DNA quantification even in the presence of a marked decrease in *E_max_* and *ΔE*. The main advantage of the proposed method is that it uses the kinetic parameters of the amplification curve using the information estimated in the inflection point of the amplification curve where the fluorescence readings are very reliable and minimally affected by noise. This is noteworthy, as the methods based on the analysis of fluorescence in the first cycles of curve growth are very sensitive to baseline evaluation and variation [37]. Moreover, the proposed method relies on the fitting of the whole fluorescence reading avoiding the serious problem of point selection by the user. Finally, the stability of this procedure is further ensured by its integration with the SOD method allowing us to detect and correct only those amplifications which showed marked different efficiencies. Taken together, these features make the proposed method very precise, as shown by the low coefficient of inter-run variation. Moreover, the improved *Cy0* method was very accurate even when overall efficiency dropped to about 60–80%. These results show the effectiveness of estimating amplification efficiency using only the parameters of the curve inflection point.

## Supporting Information

Data File S1 **Word file that describes the algebra used in** ***Cy0*** **correction.**
(DOC)

Data File S2 **Fluorescence data and fitting elaborations of standard sample amplifications (standard curve) and amplifications obtained in the presence reverse blocked primer.**
(XLS)

Data File S3 **Analysis of the variation in curve asymmetry for each amplification curve obtained using the HE and LE primer assays in presence or absence of blocked and finally in HE/LE competition setup.**
(TIF)

Data File S4 **Fluorescence data, fitting elaborations and** ***Cy0*** **calculations obtained using the freely available data set named “Competimer” reported in Ruijter** ***et al.*** [**32**] **.**
(XLS)

## References

[1] Gingeras TR, Higuchi R, Kricka LJ, Lo YM, Wittwer CT (2005) Fifty years of molecular (DNA/RNA) diagnostics. Clin Chem 51: 661–671. 15650028 10.1373/clinchem.2004.045336

[2] Nolan T, Hands RE, Bustin SA (2006) Quantification of mRNA using real-time RT-PCR. Nature Protocols 1: 1559–1582. 17406449 10.1038/nprot.2006.236

[3] VanGuilder HD, Vrana KE, Freeman WM (2008) Twenty-five years of quantitative PCR for gene expression analysis. Biotechniques 44: 619–626. 18474036 10.2144/000112776

[4] Wang Y, Barbacioru C, Hyland F, Xiao W, Hunkapiller KL, et al (2006) Large scale real-time PCR validation on gene expression measurements from two commercial long-oligonucleotide microarrays. BMC Genomics 7: 59. 16551369 10.1186/1471-2164-7-59 PMC1435885

[5] Cikos S, Bukovska A, Koppel J (2007) Relative quantification of mRNA: comparison of methods currently used for real-time PCR data analysis. BMC Mol Biol 8: 113. 18093344 10.1186/1471-2199-8-113 PMC2235892

[6] Tichopad A, Kitchen R, Riedmaier I, Becker C, Stahlberg A, et al (2009) Design and optimization of reverse-transcription quantitative PCR experiments. Clin Chem 55: 1816–1823. 19643838 10.1373/clinchem.2009.126201

[7] Bustin SA, Benes V, Garson JA, Hellemans J, Huggett J, et al (2009) The MIQE guidelines: minimum information for publication of quantitative real-time PCR experiments. Clin Chem 55: 611–622. 19246619 10.1373/clinchem.2008.112797

[8] Rutledge RG, Cote C (2003) Mathematics of quantitative kinetic PCR and the application of standard curves. Nucleic Acids Res 31: e93. 12907745 10.1093/nar/gng093 PMC169985

[9] Morrison TB, Weis JJ, Wittwer CT (1998) Quantification of low-copy transcripts by continuous SYBR Green I monitoring during amplification. Biotechniques 24: 954–958, 960, 962. 9631186

[10] Kainz P (2000) The PCR plateau phase - towards an understanding of its limitations. Biochim Biophys Acta 1494: 23–27. 11072065 10.1016/s0167-4781(00)00200-1

[11] Rutledge RG, Stewart D (2008) Critical evaluation of methods used to determine amplification efficiency refutes the exponential character of real-time PCR. BMC Mol Biol 9: 96. 18973660 10.1186/1471-2199-9-96 PMC2587475

[12] Akane A, Matsubara K, Nakamura H, Takahashi S, Kimura K (1994) Identification of the heme compound copurified with deoxyribonucleic acid (DNA) from bloodstains, a major inhibitor of polymerase chain reaction (PCR) amplification. J Forensic Sci 39: 362–372. 8195750

[13] Tichopad A, Didier A, Pfaffl MW (2004) Inhibition of real-time RT-PCR quantification due to tissue-specific contaminants. Mol Cell Probes 18: 45–50. 15036369 10.1016/j.mcp.2003.09.001

[14] Wilson IG (1997) Inhibition and facilitation of nucleic acid amplification. Appl Environ Microbiol 63: 3741–3751. 9327537 10.1128/aem.63.10.3741-3751.1997 PMC168683

[15] Rossen L, Norskov P, Holmstrom K, Rasmussen OF (1992) Inhibition of PCR by components of food samples, microbial diagnostic assays and DNA-extraction solutions. Int J Food Microbiol 17: 37–45. 1476866 10.1016/0168-1605(92)90017-w

[16] Annibalini G, Guescini M, Agostini D, Matteis RD, Sestili P, et al (2012) The expression analysis of mouse interleukin-6 splice variants argued against their biological relevance. BMB Rep 45: 32–37. 22281010 10.5483/bmbrep.2012.45.1.32

[17] Ramakers C, Ruijter JM, Deprez RH, Moorman AF (2003) Assumption-free analysis of quantitative real-time polymerase chain reaction (PCR) data. Neurosci Lett 339: 62–66. 12618301 10.1016/s0304-3940(02)01423-4

[18] Bar T, Stahlberg A, Muszta A, Kubista M (2003) Kinetic Outlier Detection (KOD) in real-time PCR. Nucleic Acids Res 31: e105. 12930979 10.1093/nar/gng106 PMC212825

[19] Tichopad A, Bar T, Pecen L, Kitchen RR, Kubista M, et al (2010) Quality control for quantitative PCR based on amplification compatibility test. Methods 50: 308–312. 20109549 10.1016/j.ymeth.2010.01.028

[20] Bar T, Kubista M, Tichopad A (2012) Validation of kinetics similarity in qPCR. Nucleic Acids Res 40: 1395–1406. 22013160 10.1093/nar/gkr778 PMC3287174

[21] Sisti D, Guescini M, Rocchi MBL, Tibollo P, D’Atri M, et al (2010) Shape Based Kinetic Outlier Detection in Real-time PCR. BMC Bioinformatics Apr 12: 186. 10.1186/1471-2105-11-186 PMC2873533 20385019

[22] Nolan T, Hands RE, Ogunkolade W, Bustin SA (2006) SPUD: a quantitative PCR assay for the detection of inhibitors in nucleic acid preparations. Anal Biochem 351: 308–310. 16524557 10.1016/j.ab.2006.01.051

[23] Goll R, Olsen T, Cui G, Florholmen J (2006) Evaluation of absolute quantitation by nonlinear regression in probe-based real-time PCR. BMC Bioinformatics 7: 107. 16515700 10.1186/1471-2105-7-107 PMC1450306

[24] Liu W, Saint DA (2002) A new quantitative method of real time reverse transcription polymerase chain reaction assay based on simulation of polymerase chain reaction kinetics. Anal Biochem 302: 52–59. 11846375 10.1006/abio.2001.5530

[25] Peirson SN, Butler JN, Foster RG (2003) Experimental validation of novel and conventional approaches to quantitative real-time PCR data analysis. Nucleic Acids Res 31: e73. 12853650 10.1093/nar/gng073 PMC167648

[26] Rutledge RG (2004) Sigmoidal curve-fitting redefines quantitative real-time PCR with the prospective of developing automated high-throughput applications. Nucleic Acids Res 32: e178. 15601990 10.1093/nar/gnh177 PMC545475

[27] Rutledge RG, Stewart D (2008) A kinetic-based sigmoidal model for the polymerase chain reaction and its application to high-capacity absolute quantitative real-time PCR. BMC Biotechnol 8: 47. 18466619 10.1186/1472-6750-8-47 PMC2397388

[28] Spiess AN, Feig C, Ritz C (2008) Highly accurate sigmoidal fitting of real-time PCR data by introducing a parameter for asymmetry. BMC Bioinformatics 9: 221. 18445269 10.1186/1471-2105-9-221 PMC2386824

[29] Guescini M, Sisti D, Rocchi MB, Stocchi L, Stocchi V (2008) A new real-time PCR method to overcome significant quantitative inaccuracy due to slight amplification inhibition. BMC Bioinformatics 9: 326. 18667053 10.1186/1471-2105-9-326 PMC2533027

[30] Guescini M, Fatone C, Stocchi L, Guidi C, Potenza L, et al (2007) Fine needle aspiration coupled with real-time PCR: a painless methodology to study adaptive functional changes in skeletal muscle. Nutr Metab Cardiovasc Dis 17: 383–393. 17482439 10.1016/j.numecd.2007.01.012

[31] Richards F (1959) A flexible growth function for empirical use. Journal of experimental Botany 10: 290–300.

[32] Ruijter JM, Pfaffl MW, Zhao S, Spiess AN, Boggy G, et al (2013) Evaluation of qPCR curve analysis methods for reliable biomarker discovery: Bias, resolution, precision, and implications. Methods 59: 32–46. 22975077 10.1016/j.ymeth.2012.08.011

[33] Chervoneva I, Li Y, Iglewicz B, Waldman S, Hyslop T (2007) Relative quantification based on logistic models for individual polymerase chain reactions. Stat Med 26: 5596–5611. 17968873 10.1002/sim.3127

[34] Rutledge RG, Stewart D (2010) Assessing the performance capabilities of LRE-based assays for absolute quantitative real-time PCR. PLoS One 5: e9731. 20305810 10.1371/journal.pone.0009731 PMC2840021

[35] Larionov A, Krause A, Miller W (2005) A standard curve based method for relative real time PCR data processing. BMC Bioinformatics 6: 62. 15780134 10.1186/1471-2105-6-62 PMC1274258

[36] Shain EB, Clemens JM (2008) A new method for robust quantitative and qualitative analysis of real-time PCR. Nucleic Acids Res 36: e91. 18603594 10.1093/nar/gkn408 PMC2504305

[37] Ruijter JM, Ramakers C, Hoogaars WM, Karlen Y, Bakker O, et al (2009) Amplification efficiency: linking baseline and bias in the analysis of quantitative PCR data. Nucleic Acids Res 37: e45. 19237396 10.1093/nar/gkp045 PMC2665230

