# Supplementary material for: Accurate and Precise DNA Quantification in the Presence of Different Amplification Efficiencies Using an Improved Cy0 Method
Source: PLoS One. 2013 Jul 8;8(7):e68481. doi: 10.1371/journal.pone.0068481 (PMC3704541; doi:10.1371/journal.pone.0068481)
Supplement: Data File S1 — Word file that describes the algebra used in Cy0 correction. (DOC) [file pone.0068481.s001.doc]

**Mathematics of Cy0 correction**

Regarding the kinetic of PCR amplification we consider the logistic model as previously reported by Chervoneva and Rutledge (1-2).

$y=\frac{F_{\max}}{1+\left(\frac{F_{\max}}{y_{0}}-1\right)\left(E_{\max}+1\right)^{-x}}$ Eq. 1

where *F_max_* is is the height of the amplification profile, *E_max_* is the starting maximal efficiency value of curve growth rate and *y_0_* is the initial amount of DNA template.

The logistic model assumes that $F_{\max}=2Y_{f}$; we can explicit $y_{f}=\frac{F_{\max}}{2}$ ,and hence Xf can be obtained as follows:

$$\frac{F_{\max}}{2}=\frac{F_{\max}}{1+\left(\frac{F_{\max}}{y_{0}}-1\right)\left(E_{\max}+1\right)^{-x_{f}}}$$

Simplifying the equation, we can rewrite:

$$\left(\frac{F_{\max}}{y_{0}}-1\right)\left(E_{\max}+1\right)^{-x_{f}}=1$$

And then:

$$\left(E_{\max}+1\right)^{x_{f}}=\left(\frac{F_{\max}}{y_{0}}-1\right)$$

Finally:

$x_{f}=\log_{\left(E_{\max}+1\right)}\left(\frac{F_{\max}}{y_{0}}-1\right)$ Eq. 2

Then we calculate the slope in inflection point deriving versus x:

$\frac{dy}{dx}=F_{\max}\frac{1}{\left[1+\left(\frac{F_{\max}}{y_{0}}-1\right)\left(E_{\max}+1\right)^{-x}\right]^{2}}\left(\frac{F_{\max}}{y_{0}}-1\right)\left(E_{\max}+1\right)^{-x}\ln\left(E_{\max}+1\right)$ Eq. 3

Then substituting in *dy/dx* (Eq. 3) the *x_f_* value (Eq. 2) we obtained the slope in the inflection point (*m*):

$$m=F_{\max}\frac{1}{\left[1+\left(\frac{F_{\max}}{y_{0}}-1\right)\left(E_{\max}+1\right)^{-\log_{\left(E_{\max}+1\right)}\left(\frac{F_{\max}}{y_{0}}-1\right)}\right]^{2}}\left(\frac{F_{\max}}{y_{0}}-1\right)\left(E_{\max}+1\right)^{-\log_{\left(E_{\max}+1\right)}\left(\frac{F_{\max}}{y_{0}}-1\right)}\ln\left(E_{\max}+1\right)$$

Further simplifying:

$$m=F_{\max}\frac{1}{\left[1+\left(\frac{F_{\max}}{y_{0}}-1\right)\left(\frac{F_{\max}}{y_{0}}-1\right)^{-1}\right]^{2}}\left(\frac{F_{\max}}{y_{0}}-1\right)\left(\frac{F_{\max}}{y_{0}}-1\right)^{-1}\ln\left(E_{\max}+1\right)$$

then:

$$m=F_{\max}\frac{1}{\left[1+1\right]^{2}}\ln\left(E_{\max}+1\right)$$

And :

$$m=F_{\max}\frac{\ln\left(E_{\max}+1\right)}{4}$$

Finally:

$$\frac{m}{y_{f}}=\frac{\ln\left(E_{\max}+1\right)}{2}$$

In order to calculate the starting DNA quantity ($y_{0}$), the logistic function (Eq. 1) can be rewritten as follows:

$$y_{0}=\frac{F_{\max}}{1+\left(\frac{F_{\max}}{y}-1\right)\left(E_{\max}+1\right)^{x}}$$

In the inflection point, substituting $F_{\max}=2Y_{f}$:

$$y_{0}=\frac{2y_{f}}{1+\left(\frac{2y_{f}}{y_{f}}-1\right)\left(E_{\max}+1\right)^{x_{f}}}$$

that could be simplified in:

$y_{0}=\frac{2y_{f}}{1+\left(E_{\max}+1\right)^{x_{f}}}$ Eq. 4

If we consider the Eq. 4 for two different amplification reactions (A and B) showing the same starting DNA template ($y_{0}$), but different initial amplification efficiencies (*E_max_*); we can consider the following equivalence:

$$\frac{y_{f_{A}}}{1+\left(E_{\max_{A}}+1\right)^{x_{f_{A}}}}=\frac{y_{f_{B}}}{1+\left(E_{\max_{B}}+1\right)^{x_{f_{B}}}}$$

Then if we standardize for one flex ordinate (for example *y_fA_*), we can obtain:

$$\frac{1}{1+\left(E_{\max_{A}}+1\right)^{x_{f_{A}}}}=\frac{\frac{y_{f_{B}}}{y_{f_{A}}}}{1+\left(E_{\max_{B}}+1\right)^{x_{f_{B}}}}$$

The denominator values are usually higher than 1000 since, in most cases, (*E_max_*+1)>1.7 and x*_f_*>10-12. Therefore it is possible to introduce the following approximation:

$$1+\left(E_{\max}+1\right)^{x_{f}}\simeq\left(E_{\max}+1\right)^{x_{f}}$$

This approximation entails a maximal error <0.001. Then

$$\frac{1}{\left(E_{\max_{A}}+1\right)^{x_{f_{A}}}}=\frac{\frac{y_{f_{B}}}{y_{f_{A}}}}{\left(E_{\max_{B}}+1\right)^{x_{f_{B}}}}$$

applying logarithmic transformation, we obtain:

$x_{f_{A}}\ln\left(E_{\max_{A}}+1\right)=x_{fB}\ln\left(E_{\max_{B}}+1\right)-\ln\frac{y_{f_{B}}}{y_{f_{A}}}$ Eq. 5

but

$\frac{2m}{y_{f}}=\ln\left(E_{\max}+1\right)$ Eq. 6

Substituting the Eq. 6 in the Eq. 5, we obtain:

$$x_{f_{A}}\frac{2m_{A}}{y_{f_{A}}}=x_{f_{B}}\frac{2m_{B}}{y_{f_{B}}}-\ln\frac{y_{f_{B}}}{y_{f_{A}}}$$

And finally:

$$x_{f_{A}}=\left(x_{f_{B}}\frac{2m_{B}}{y_{f_{B}}}-\ln\frac{y_{f_{B}}}{y_{f_{A}}}\right)\frac{y_{f_{A}}}{2m_{A}}$$

It is possible to consider *x_f_* proportional to *Cy0* value (*x_f_* α *Cy0*).

$$Cy0_{_{A}}=\left(Cy0_{_{B}}\frac{2m_{B}}{y_{f_{B}}}-\ln\frac{y_{f_{B}}}{y_{f_{A}}}\right)\frac{y_{f_{A}}}{2m_{A}}$$
