# Supplementary figures and images for: Accurate and Precise DNA Quantification in the Presence of Different Amplification Efficiencies Using an Improved Cy0 Method
Source: PLoS One. 2013 Jul 8;8(7):e68481. doi: 10.1371/journal.pone.0068481 (PMC3704541; doi:10.1371/journal.pone.0068481)

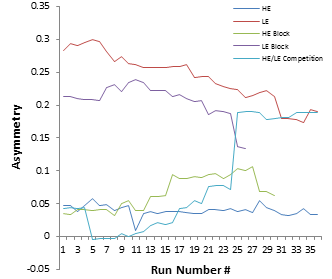

Supplement: Data File S3 — Analysis of the variation in curve asymmetry for each amplification curve obtained using the HE and LE primer assays in presence or absence of blocked and finally in HE/LE competition setup. (TIF) [file pone.0068481.s003.tif]
